# Repercussions of sleep quality on the P300 auditory evoked potential in older adults

**DOI:** 10.1590/1980-5764-DN-2024-0271

**Published:** 2025-09-01

**Authors:** Letícia Pimenta Costa-Guarisco, Elynaide Cínthia da Silva Julião, Ana Julia de Lima Bomfim

**Affiliations:** 1 Universidade Federal de São Carlos, Centro de Ciências Biológicas e da Saúde, Departamento de Gerontologia, São Carlos SP, Brazil.; 2 Universidade de São Paulo, Faculdade de Medicina de Ribeirão Preto, Programa de Pós-Graduação em Saúde Mental, Ribeirão Preto SP, Brazil.

**Keywords:** Aged, Cognition, Sleep Quality, Event-Related Potentials, P300, Idoso, Cognição, Qualidade do Sono, Potenciais Evocados P300

## Abstract

P300 is a long-latency evoked potential that takes place around 300 milliseconds after the onset of a relevant stimulus. It can be assessed by measuring the latency and amplitude of the P3 wave, which are associated with information processing time and attention level, respectively.

**Objective:**

To investigate the association between sleep quality and latency and amplitude of the P300 wave in aged individuals.

**Methods:**

A quantitative and observational cross-sectional study was conducted with 28 older adults, excluding those with cognitive impairments or hearing loss. Sociodemographic data, P300 test latency and amplitude and the Mini Sleep Questionnaire were used, which defined two study groups: good sleep and altered sleep.

**Results:**

The results indicated that the group with altered sleep quality presented lower P3 wave amplitudes in the Cz, Fz and Pz channels (p=0.07; p=0.00; p=0.01, respectively). No significant differences were observed in the P300 wave latency between the groups. In the correlation test, it was noticed that the more sleep altered, the lower the P3 wave amplitude in the Fz channel (rho=-0.46, p=0.02).

**Conclusion:**

P300 amplitude was sensitive to changes in sleep quality, suggesting a decline in attention without a corresponding effect on the information processing speed. There is a relationship between amplitude of the P300 wave and sleep quality in aged people, suggesting that poor sleep quality exerts negative impacts on cognitive performance.

## RESUMO

O P300 é um potencial evocado de longa latência que acontece cerca de 300 milissegundos após o início de um estímulo relevante. Ele pode ser avaliado por meio das medidas de latência e amplitude da onda P3, relacionadas ao tempo de processamento da informação e ao nível de atenção, respectivamente.

**Objetivo:**

Verificar a associação e relação entre a qualidade do sono e a latência e amplitude da onda P300 em pessoas idosas.

**Métodos:**

Foi realizado um estudo observacional quantitativo de corte transversal com 28 pessoas idosas, sem alterações cognitivas ou perdas auditivas. Foram utilizados os dados sociodemográficos, latência e amplitude do teste P300 e o Miniquestionário do Sono, que definiu dois grupos de estudo: com sono bom e com sono alterado.

**Resultados:**

Os resultados demonstraram que o grupo com qualidade do sono alterada apresentou menor amplitude da onda P3 nos canais Cz, Fz e Pz (p=0,07; p=0,00; p=0,01; respectivamente). Não houve diferença significativa na latência da onda P300 entre os grupos. No teste de correlação, observou-se que, quanto mais alterado o sono, menor a amplitude da onda P3 no canal Fz (rho=-0,46, p=0,02).

**Conclusão:**

A amplitude do P300 foi sensível às alterações na qualidade do sono, o que sugere diminuição da atenção, sem interferência na velocidade do processamento da informação. Dessa forma, existe relação entre a amplitude da onda P300 e a qualidade do sono em pessoas idosas, sugerindo que a má qualidade do sono impacta negativamente o desempenho cognitivo.

## INTRODUCTION

Aging is an intrinsic, multifaceted and individualized process that is significantly influenced by lifestyle habits, during which morphological and physiological changes take place throughout the body^1^. From this perspective, as individuals age, changes in their sleep pattern occur as part of the natural aging process. Although the total sleep hours required may remain stable, a decrease in sleep efficiency and depth is commonly observed. Older adults tend to spend more time in lighter sleep stages and less time in deep sleep, resulting in more frequent awakenings throughout the night^2^. These events may be associated with a wide range of factors, including physiological changes, underlying health conditions and psychological and environmental factors^3^. However, sleep disturbances can lead to adverse health outcomes, including heart disease, anxiety disorders, depression, falls, accidents, cognitive impairment, decreased quality of life, and even death^4^.

Sleep consists of two stages: rapid eye movement (REM) sleep, characterized by such eye movements, intense brain activity and muscle relaxation; and non-rapid eye movement (NREM) sleep, which consists of three progressive stages ranging from light sleep to deep sleep. During NREM sleep, memory consolidation and physical recovery take place, while REM sleep plays a crucial role in emotional and cognitive processing, as well as being associated with vivid dreaming. These stages alternate throughout the night in successive cycles, promoting restorative, high-quality sleep^3^.

Good sleep quality is characterized by the ability to maintain continuous and restorative sleep, which includes achieving an adequate deep-REM sleep proportion, experiencing minimal disruptions throughout the night and waking up feeling rested^5^.

According to the Brazilian Sleep Association^6^, older adults generally go to bed earlier than younger adults, enjoying more than seven consecutive hours of good-quality sleep. Daytime naps are common, while most of the sleep hours are at night. This pattern is typical and should not be regarded as a sleep disorder. However, factors such as low physical activity levels and social isolation (which are common during retirement) can affect sleep.

Poor sleep quality is one of the most common complaints among older adults and is a condition that can adversely affect health and individual well-being. Sleep is essential for promoting rest, physical and mental recovery, cognitive function and emotional well-being. Some studies suggest that poor sleep quality is associated with an increased risk of cognitive decline^4,7,8^. Aspects such as sleep duration, daytime sleepiness, naps and total sleep hours exert an influence on cognition and may lead to impaired cognitive performance in older adults^8^.

The long-latency auditory evoked potential (P300) is an electrophysiological test that allows identifying potential dysfunctions and/or alterations in the central auditory nervous system (CANS) through an objective measure of cognitive processes, such as attention level and information processing speed, in relation to an auditory task involving attention, discrimination, stimulus detection and memory^9^. The P300 wave takes place approximately 300 milliseconds after the onset of a relevant stimulus for the task and can be elicited in response to visual, auditory or somatosensory stimuli^10^. Two variables are used to quantify P300: wave latency, which reflects the time required for information processing; and amplitude, which is associated with the attention level^11^.

Few studies have investigated the relationship between sleep disorders and cognitive changes using the P300 test. One of them demonstrated that sleep deprivation caused by the obstructive sleep apnea syndrome (OSAS) leads to cognitive deterioration, as evidenced by changes in latency and amplitude of the P300 wave^12^. Another study, also related to OSAS, suggested using electrophysiological tests for cognitive assessment^13^. Both studies highlight the P300 wave as an essential tool for investigating cognitive changes related to sleep disorders.

It is believed that, by influencing cognitive performance, sleep quality may affect attention levels and the auditory information processing speed and, consequently, the P300 responses. The objective of this study was to examine the relationship between sleep quality and cognitive performance (P300) in older adults.

## METHODS

This is a cross-sectional and observational study with a quantitative approach to the data. The project was approved by the Research Ethics Committee of the affiliated university (Certificate of Presentation for Ethical Appreciation—CAAE: 69137123.4.0000.5504; approval number: 6,482,515). All participants signed the free and informed consent form as required by Resolution No. 466/12 of the National Health Council. Upon agreement, a date and time for the assessments was scheduled, with all evaluations conducted between November 2023 and February 2024 at the Gerontology Department belonging to the Universidade Federal de São Carlos (UFSCar).

To carry out data collection, community-dwelling aged individuals were invited in person, either by close acquaintances or through referral. The study inclusion criterion was individuals aged at least 60 years old. People with bilateral hearing loss at 1,000 and 2,000 Hz at 40 dB HL and those showing cognitive impairment based on the Mini-Mental State Examination (MMSE) were excluded. A total of 36 individuals were assessed, of whom one was excluded due to hearing impairment and seven due to cognitive alterations. Therefore, the final sample consisted of 28 aged individuals.

### Evaluation instruments

#### Sociodemographic data

A sociodemographic assessment was conducted with each participant, during which data were collected on age (in full years old), gender (female or male), schooling level (years of study), marital status (whether or not they have a partner) and occupation (student, worker or housewife).

#### Mini-Mental State Examination

The participants’ cognitive function was assessed using the Mini-Mental State Examination (MMSE), a neuropsychological test for screening cognitive decline that was developed by Folstein et al.^14^ and translated and adapted for the Brazilian context by Bertolucci et al.^15^. The MMSE cutoff scores are set as follows: 13 points or less for illiterate individuals; 18 points for individuals with low schooling (one to eight years of studies); and 26 points for individuals with high schooling levels (more than eight years of study). The subjects whose scores fell below the cutoff for their schooling level were excluded from the sample.

#### Mini Sleep Questionnaire

The Mini Sleep Questionnaire assesses the frequency of sleep-related complaints based on two factors: difficulty falling asleep and overall sleep quality. The test has ten questions, each with seven answer options. The total score ranges from 10 to 70 points, with higher values indicating poorer sleep quality. The individuals were categorized into two groups: good sleep (up to 24 points) and altered sleep (25 points or more)^16,17^.

#### Long-latency auditory evoked potential (P300)

The P300 auditory evoked potential was obtained using a Neurosoft instrument, model Neuron-Spectrum-4/EPM. To ensure precise electrode placement on the head, an elastic textile cap from MCScap was used, secured with a chin strap. The cap size was determined based on head circumference. A set of sintered Ag/AgCl electrodes with individual connectors was used, model MCScap-E.

Before attaching the electrodes, the areas where they would be placed were cleaned with alcohol and gauze. An abrasive gel was also applied to prepare the skin, with the aim of reducing skin impedance and, consequently, improving the test results. The electrodes were then positioned and the conductive gel was applied to improve conductivity and ease wave transmission during the test.

The contact electrodes were attached to the frontal (Fz), central (Cz) and parietal (Pz) scalp regions and the ground electrode was placed at the forehead (Fpz), following the International 10/20 System^18^. The reference electrodes (interconnected) were placed on the right (A2) and left (A1) earlobes.

All assessments were conducted during the morning, in a quiet environment with electrical grounding and controlling for acoustic and electrical interferences during the test. To perform the P300 test, the participants were seated comfortably in a chair and instructed to keep their eyes open, focusing on a fixed point in front of them. Non-verbal auditory stimuli were used and the sequence of stimuli was delivered binaurally through TA-01 model headphones. The stimulus intensity ranged from 80 to 90 dB and could be adjusted according to the participants’ sensitivity and comfort with the sound. The auditory evoked potential was recorded using the *oddball* paradigm, based on the discrimination of rare stimuli, presented randomly 20% of the time at a 2,000 Hz frequency, in contrast with frequent stimuli, presented 80% of the time at a 1,000 Hz frequency. The participants were instructed to press a button with their dominant hand in response to the rare stimulus. Considering the time required for cleaning and electrode placement, the test lasted approximately 15 to 20 minutes.

After recording the waves generated by the long-latency auditory potential, the P1, N1, P2, N2 and P3 waves were identified in the Cz, Pz, and Fz channels and the latency and amplitude values corresponding to the P300 waves were recorded. Amplitude and latency measures were considered as the outcomes. Latency was measured by identifying the maximum positive amplitude within the 250-500 ms interval following stimulus onset. The amplitude analysis was performed based on the absolute amplitude (baseline-P300).

The analysis of this potential included waveform analysis, which examines wave morphology, as well as the assessment of amplitude and latency measurements in the Fz, Cz and Pz channels. In this context, the P1, N1, P2, N2 and P300 components were manually identified and labeled. The P1, N1, P2 and N2 components were identified in the trace corresponding to the frequent stimulus, while the P300 component was identified in the trace corresponding to the rare stimulus.

Regarding the P1, N1, P2 and N2 components, the first three waves observed in the sequence were considered, presenting positive-negative-positive-negative polarity, respectively, replicated both in the ‘frequent’ and in the ‘rare’ trace between 60 and 300 ms.

The method used to identify P300 involved locating the largest positive wave following the P1, N1, P2 and N2 components, after presentation of the rare stimulus, which corresponds to the maximum wave amplitude point^19^. The amplitude analysis was performed using the absolute amplitude (baseline-P300).

### Data analysis

The data collected were entered into an Excel spreadsheet, with the participants’ names replaced by numbers to ensure anonymity. The data were then exported to the Statistical Package for the Social Sciences (SPSS), version 21.0, where they were analyzed. Descriptive statistics were performed to describe the sample profile, including position and dispersion measures (mean, standard deviation, minimum and maximum values and median) for the continuous variables. Frequency tables with absolute (n) and percentage (%) values were created for the categorical variables.

Two study groups were defined based on the Mini Sleep Questionnaire results: good sleep group and altered sleep group. The groups were compared based on age, schooling level, and P300 wave latency and amplitude. Given the sample size, the Mann-Whitney test was used, preliminarily assuming data non-normality. In addition, the correlation between the data was assessed using Spearman’s rank correlation coefficient. The significance level for the statistical tests was set at 5% (p≤0.05).

## RESULTS

Table 1 shows the sociodemographic data corresponding to the sample (N=28). The mean age was 66.75 years old, ranging between 60 and 87. The participants were predominantly women, most of them with a partner, over eight years of studies, and either retired or housewives.

**Table 1:** Sociodemographic data corresponding to the sample (N=28). São Carlos, São Paulo, Brazil, 2024.

Characteristics	Frequency (%)	Minimum	Maximum	Mean	SD
Gender					
Female	23 (82.1)	-	-	-	-
Male	5 (17.9)	-	-	-	-
Age	-	60	87	66.75	6.29
Schooling (years)	-	2	25	11.71	6.55
0–4	8 (28.6)	-	-	-	-
5–8	2 (7.1)	-	-	-	-
>8	18 (64.3)	-	-	-	-
Occupation					
Worker/Student	7 (25.0)	-	-	-	-
Retired/Housewife	21 (75.0)	-	-	-	-
Has a partner					
Yes	16 (57.1)	-	-	-	-
No	12 (42.9)	-	-	-	-

Abbreviation: SD, standard deviation.

Table 2 presents the results of the relationship between sleep quality and age and schooling, as well as the P300 latency and amplitude measures. The groups with good and altered sleep were similar in terms of age and schooling. The good sleep group had ages from 61 to 81 years old, while the altered sleep group had ages from 61 to 87, both with the same median (66). When comparing the P300 results, it was observed that the group with altered sleep had a lower wave amplitude when compared to the group with good sleep, particularly in the Fz and Pz channels. No statistically significant differences were found in the P300 wave latency between the groups across any of the channels.

**Table 2:** Relationship between sleep quality and age and schooling, as well as the P300 latency and amplitude measures. São Carlos, São Paulo, Brazil, 2024.

	Good sleep (N=15)			Altered sleep (N=13)			
	N (%)	Mean	SD	N (%)	Mean	SD	p-value
Gender							
Female	12 (80)	-	-	11 (84)	-	-	-
Male	3 (20)	-	-	2 (15)	-	-	-
Age	15	65.80	4.39	13	66.76	7.20	0.92
Schooling	15	10.73	7.22	13	12.85	5.77	0.21
P300 latency							
Cz	14	364.85	43.97	11	364.63	74.25	0.70
Fz	13	361.76	46.02	11	355.27	57.46	0.68
Pz	13	371.84	40.48	11	352.54	61.21	0.13
P300 amplitude							
Cz	14	8.67	4.23	11	5.12	4.04	0.07*
Fz	13	8, 94	3.06	11	5.15	3.41	0.00*
Pz	13	12.51	4.60	11	7.68	3.69	0.01*

Note:*p<0.05.

Table 3 shows the correlation between sleep quality and the P300 latency and amplitude measures in the Cz, Fz and Pz channels. A moderate negative correlation was observed between sleep quality and P300 amplitude in the Fz channel, indicating that the poorer the sleep quality (the higher the score), the lower the P300 wave amplitude.

**Table 3:** Correlation between the “sleep quality” and “age” variables, as well as “P300 wave amplitude and latency”.

		Age	Schooling	Latency			Range		
				Cz	Fz	Pz	Cz	Fz	Pz
Sleep quality	Correlation	-0.02	0.30	-0.08	-0.07	-0.32	-0.29	-0.46	-0.30
	p-value	0.88	0.117	0.69	0.73	0.12	0.15	0.02*	0.15
	N	28	28	25	24	24	25	24	24

## DISCUSSION

Sleep is essential for regulating brain activity and maintaining cognitive functions, particularly in older adults, who are at greater risk of cognitive decline. During sleep, critical processes such as synaptic activity modulation and memory consolidation take place, both of which are vital for learning and reasoning^20^. Additionally, REM sleep is essential to reorganize synaptic connections, integrating new information with prior knowledge. This process is vital for brain plasticity and for the formation of long-term memories^21^. Feld and Born^22^ suggest that memory consolidation during sleep not only stores new memories but also reorganizes and integrates them with prior knowledge, which in turn enhances cognitive activities such as creativity and problem-solving. Supporting these ideas, the current study showed that sleep quality is related to cognitive potential in older adults.

Event-related potentials (ERPs) represent a crucial non-invasive approach for studying information processing and cognitive functions in the brain. The P300 brain wave is a component assessed through amplitude measures that reflect task-related attentional resources and latency measures, which are characterized by the reaction time to a given stimulus^23,24^. If repeatedly presented, a standard stimulus forms a well-established memory in the brain. In contrast, a target stimulus, which appears less frequently, tends to have a less consolidated memory. When a target stimulus is detected, the memory needs to be reviewed and updated. For this update to take place, attentional resources are required to identify the target stimulus and maintain the standard stimulus memory active^23^. When reacting to the target stimulus, cortical activity is recorded through P300 wave composition, which takes place approximately 300 ms after perception of the stimulus.

In the current study, cognitive potential was assessed using the P300 electrophysiological test, where waves were captured through electrodes placed on the skull surface in the frontal (Fz), central (Cz) and occipital (Pz) regions. Using three active electrodes provides an additional resource to analyze the long-latency auditory evoked potential (P300), as the precise cortical location where cognitive potential is elicited remains uncertain^24^.

No differences were found in P300 wave latency between the good sleep and altered sleep groups. However, it was observed that the altered sleep group presented a lower wave amplitude when compared to the good sleep group, particularly in the Fz and Pz channels. This result suggests that older individuals with altered sleep quality have fewer attentional resources available for task performance, thus exhibiting poorer cognitive performance as measured by P300^25^.

The results of the current study partially corroborate the literature, which associates not only P300 amplitude with sleep quality but also latency, emphasizing how alterations in sleep patterns can exert impacts on cognitive performance. Reflecting the time required for information processing, P300 latency tends to be longer in individuals with sleep deprivation, leading to reduced cognitive efficiency, such as sustained attention and working memory, particularly in aged populations. Associated with attention allocation and cognitive involvement, amplitude is generally reduced in individuals with insufficient or fragmented sleep, reflecting a diminished capacity to respond to external stimuli and deficits in executive functions such as inhibitory control and cognitive flexibility^26,27^.

In a study conducted with a young population, individuals with less sleep or poorer sleep quality had greater latency and reduced P300 amplitude, indicating that sleep quality exerts significant impacts on cognitive performance^27^.

The study by Pavarini et al.^11^ supports the idea that cognitive functions, which can be exacerbated by poor sleep quality, are reflected in the P300 measurements. These findings are consistent with the literature, which demonstrates that both acute and chronic sleep deprivation affect cognitive functions.

Acute sleep deprivation takes place when an individual stays awake for more than 24 hours, leading to immediate declines in attention and mental functioning, along with a strong feeling of sleepiness. On the other hand, chronic sleep deprivation, which makes up the basis of our study, occurs when a person consistently sleeps less than necessary over several days or weeks, resulting in gradual attention and cognitive performance declines, although the subjective feeling of sleepiness may not be as intense as in acute sleep deprivation cases^26^. Nonetheless, sleep deprivation not only reduces brain activity in attention-related areas but also disrupts connectivity with the cortex during cognitive tasks. This leads to behavioral challenges, such as difficulty focusing on specific stimuli and maintaining attention over time^28^.

The hypothalamus, brainstem, thalamus, cerebral cortex and pineal gland are Central Nervous System areas that work together to regulate sleep. The subcortical hypothalamus interacts with multiple brain regions (including the prefrontal cortex) through intricate neural circuits and release of hormones. These interactions exert an influence on sleep patterns. Located in the frontal part of the brain, the prefrontal cortex plays a crucial role in cognition, planning, decision-making and social behavior, and is strongly influenced by sleep quality^29^. This finding may explain the correlation observed in the P300 wave amplitude, particularly in the frontal (Fz) channel, in individuals with poorer sleep quality.

A number of studies reinforce the link between poor sleep quality and decline in cognitive functions. In the study by Yaman et al.^30^, patients with idiopathic hypersomnia showed altered sleep quality and a significant reduction in the P300 wave amplitude, reflecting deficits in attentional resources and poorer cognitive performance. These results highlight the direct relationship between poor sleep quality and decline in cognitive functions, particularly regarding sustained attention. In the study by Ray et al.^31^, 24 sleep deprivation hours led to a significant decrease in the P300 wave amplitude, indicating reduced attentional resources and impaired cognitive functions. In the article by Nada et al.^13^, the authors investigated the use of electrophysiological tests (including P300) to detect cognitive impairment in patients with obstructive sleep apnea-hypopnea syndrome (OSAHS). The findings revealed that, in addition to prolonged latency, the P300 wave amplitude was also significantly reduced in these patients. This decrease in P300 amplitude indicates a reduced ability to allocate attentional resources, suggesting that poor sleep quality in OSAHS patients negatively affects cognitive function.

In the current study, it is important to note that there was no median difference between the groups regarding the participants’ age and schooling, as both groups were comprised by aged individuals with high schooling levels. It is well established that age and education exert significant impacts on cognitive processes, particularly in relation to cognitive reserve in older adults^11^. Thus, having groups with equivalent age and schooling levels was a favorable factor for the analysis, as it allowed for a more accurate comparison of the P300 cognitive variable between the groups, minimizing the potential influence of age and schooling on the results. On the other hand, no correlation was found between the P300 components and the individuals’ age or schooling level, which contradicts the existing literature.

The gender variable was not studied due to lack of male representation in the sample, which can be considered a study limitation. Future studies should aim at increasing the sample size and including a more balanced gender distribution.

In addition, it is important to note that the convenience sample comprised by 28 participants may have limited the ability to produce more robust results. These factors should be considered when interpreting the findings, as the sample is not representative of the broader aged population. Furthermore, the exclusive use of the Mini Sleep Questionnaire, which primarily assesses insomnia and hypersomnia, fails to record all aspects inherent to sleep quality. For future studies, it is recommended that more comprehensive tools be used to assess sleep quality, as they might provide a clearer understanding and greater precision regarding the relationship between sleep and cognition in aged populations.

The results of this study indicate a relationship between poor sleep quality and cognitive impairment in the elderly. Considering the effects of sleep disorders on cognitive function, it is important to implement strategies for promoting sleep hygiene and cognitive monitoring in this population segment. In addition, future research should adopt longitudinal approaches to assess the impact of changes in sleep quality over time on cognition.

Some variations in the P300 latency and amplitude values between this study and others may be due to factors such as differences in equipment, participants’ attention, age, time of day when the test was conducted and stimulus counting method used, among others.

## Data Availability

The datasets generated and/or analyzed during the current study are not publicly available due to [ethical/legal/privacy] restrictions but are available from the corresponding author upon reasonable request.
